# The link between gene duplication and divergent patterns of gene expression across a complex life cycle

**DOI:** 10.1093/evlett/qrae028

**Published:** 2024-07-02

**Authors:** James G DuBose, Jacobus C de Roode

**Affiliations:** Department of Biology, Emory University, Atlanta, GA, United States; Department of Biology, Emory University, Atlanta, GA, United States

**Keywords:** life history evolution, evolutionary genomics, gene expression, mutations

## Abstract

The diversification of many lineages throughout natural history has frequently been associated with evolutionary changes in life cycle complexity. However, our understanding of the processes that facilitate differentiation in the morphologies and functions expressed by organisms throughout their life cycles is limited. Theory suggests that the expression of traits is decoupled across life stages, thus allowing for their evolutionary independence. Although trait decoupling between stages is well established, explanations of how said decoupling evolves have seldom been considered. Because the different phenotypes expressed by organisms throughout their life cycles are coded for by the same genome, trait decoupling must be mediated through divergence in gene expression between stages. Gene duplication has been identified as an important mechanism that enables divergence in gene function and expression between cells and tissues. Because stage transitions across life cycles require changes in tissue types and functions, we investigated the potential link between gene duplication and expression divergence between life stages. To explore this idea, we examined the temporal changes in gene expression across the monarch butterfly (*Danaus plexippus*) metamorphosis. We found that within homologous groups, more phylogenetically diverged genes exhibited more distinct temporal expression patterns. This relationship scaled such that more phylogenetically diverse homologous groups showed more diverse patterns of gene expression. Furthermore, we found that duplicate genes showed increased stage-specificity relative to singleton genes. Overall, our findings suggest an important link between gene duplication and the evolution of complex life cycles.

## Introduction

Many groups of organisms undergo extensive morphological and ecological shifts throughout their life cycles. These shifts appear gradual in some organisms, as seen in the relatively continuous development from infant to adult in primates. However, these shifts seem more complex in many other organisms; a larva first must transition into an intermediate pupal stage before restructuring its morphology into the form of a butterfly. Changes in life cycle complexity have been associated with the diversification of many taxa throughout natural history (Reiss, 2002; Wheeler et al., 2001). Despite nearly a century of interest in the evolution of complex life cycles, we still lack a general understanding of the mechanisms that facilitate divergence in the morphologies and functions expressed by organisms throughout their lives.

This gap in our understanding can be partially attributed to the view that complex life cycles are divided into stages that are discrete, which is the central assumption made in foundational theoretical work (Istock, 1967; Moran, 1994). While this assumption can capture the punctual ecological and developmental dynamics exhibited by organisms that are considered to have complex life cycles, it has also generated the dichotomy that organisms either do or do not have complex life cycles (Moran, 1994). However, considering life cycle complexity as a continuous spectrum has the potential to provide more basic insight into the processes that facilitate life cycle evolution. It is apparent that the transition from one life stage to the next requires continuous changes in the relative abundance, activity, or placement of different cells and tissues (Haldane, 1932). Therefore, we propose that from an organismal perspective, life cycle evolution can be more fundamentally described by the body of theory concerning the evolution of cell and tissue differentiation, which focuses on describing the mechanisms that facilitate evolutionary change in gene function and patterns of expression.

The adaptive decoupling hypothesis is the most prominent explanation for the evolution of complex life cycles, and is an extension of an earlier hypothesis that different life stages adapt independently to the niches they occupy (Istock, 1967; Moran, 1994). The adaptive decoupling hypothesis elaborates that complex life cycles allow different stages to independently respond to natural selection by genetically decoupling the development of their traits (Moran, 1994). This hypothesis predicts that genetic variation should generate phenotypic variation in certain life stages but not others. Many studies have found results consistent with this prediction (Aguirre et al., 2014; Fellous & Lazzaro, 2011; Goedert & Calsbeek, 2019; Schott et al., 2022), and more recent studies have elucidated variation in gene expression between stages as the likely driver of said genetic independence (Collet et al., 2023; Herrig et al., 2021). However, the predictions made by the adaptive decoupling hypothesis are limited to descriptions of extant signatures of decoupled traits, which fails to provide a mechanism that explains how trait decoupling evolves. We propose that the evolution of trait decoupling can be explained by the mechanisms already established in the evolution of cell and tissue differentiation because transitions between life stages are driven by continuous turnover in cell and tissue types and functions.

Gene duplication is the best described mechanism that generates evolutionary change in patterns of gene expression between cells and tissues (Gu et al., 2002; He & Zhang, 2005; Huminiecki & Wolfe, 2004; Li et al., 2005). However, other nonexclusive mechanisms, such as regulatory network evolution, are likely at play but have been more challenging to empirical study (Teichmann & Babu, 2004; Wagner, 2001; Zhang et al., 2004). Gene duplication can be generated through unequal crossing over, retrotransposition, and chromosomal duplication and provides a rich source of genetic variation that can facilitate major evolutionary change (Ohno & Susumu, 1970; Zhang, 2003). Hypotheses concerning the evolution of duplicate genes share key similarities with the adaptive decoupling hypothesis. For example, the neofunctionalization hypothesis suggests that retention of the ancestral function in one copy alleviates selective constraints on the other copy, allowing it to develop novel functions more efficiently (Ohno & Susumu, 1970). However, a role of neutral evolution in generating functional divergence between duplicate genes has also been described (Force et al., 1999; He & Zhang, 2005), thus offering a mechanism by which traits could diverge between stages that more comprehensively accounts for the processes that drive evolutionary change. More generally, the idea that complexity is added to a genome through gene duplication is well established (Lynch & Conery, 2003; Martin, 1999; Ohno & Susumu, 1970), and empirical evidence for gene duplication resulting in more complex phenotypes has been documented in a variety of taxa (Chen et al., 2023; Leite et al., 2018; Rivera et al., 2010; Tian et al., 2008). Therefore, investigating the link between gene duplication and life cycle evolution has the potential to broaden our understanding of how biological complexity evolves.

**Figure 1.**
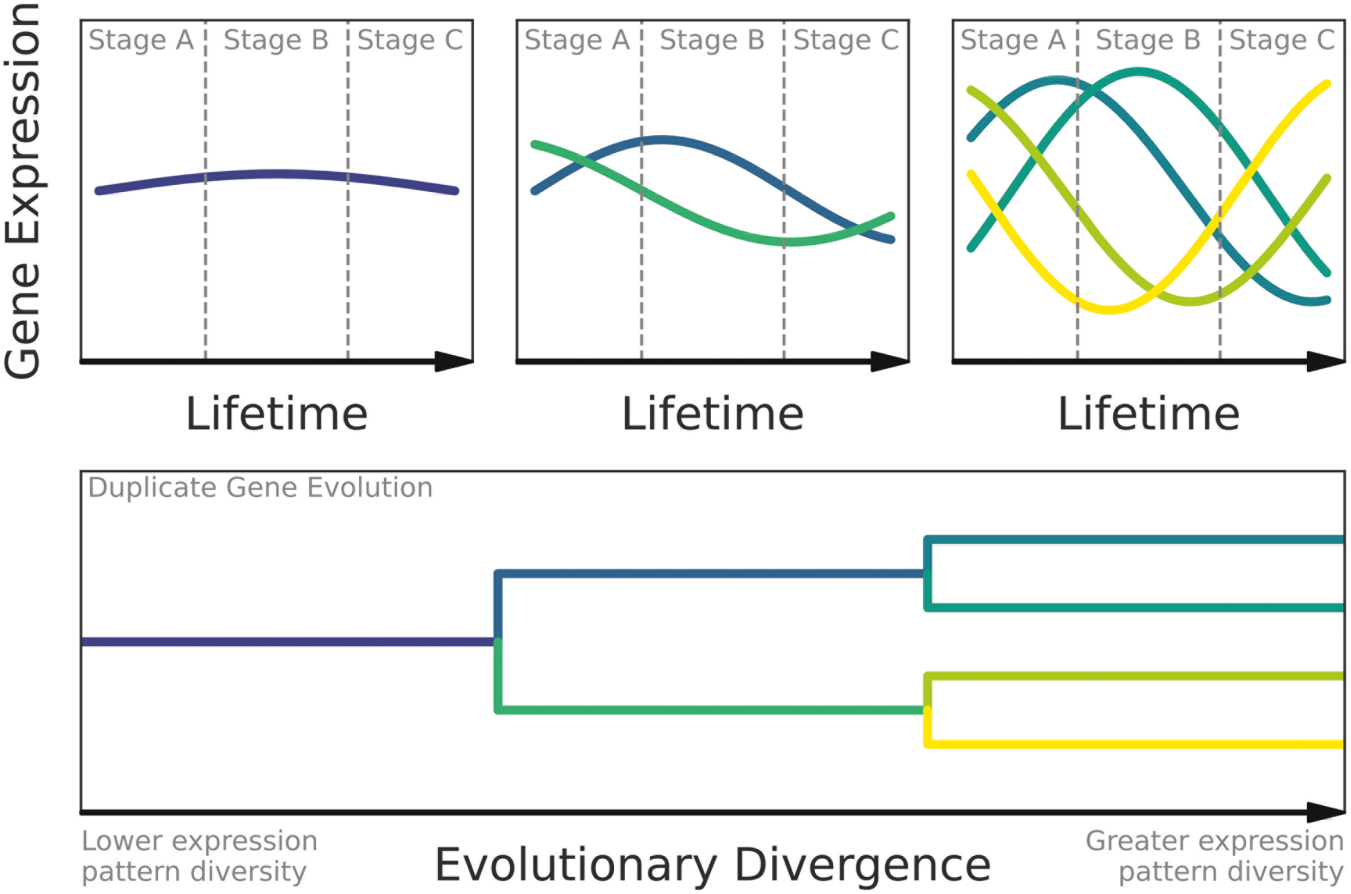
A conceptual diagram showing the hypothesized mechanism of how duplicate gene evolution could lead to divergence in gene expression (and consequently phenotypes) between perceived stages in a complex life cycle. Initially, a given gene has an expression pattern that is relatively uniform throughout an organism’s lifetime. After duplication, the expression patterns of each copy tend to diverge and become more stage-specific. After additional duplication and divergence, expressions tends to diverge and specify even more between copies. This makes the expression at each stage substantially more distinct from other stages, which would result in greater phenotypic divergence between stages if the duplicates functionally diverged as well. This diagram does not show all possible fates of duplicate genes.

Although there are several nuances to predicting the relationship between sequence evolution and expression pattern evolution in duplicate genes, the general expectation is that the evolution of duplicate genes leads to more divergent and (stage) specific expression patterns (Huminiecki & Wolfe, 2004; Li et al., 2005). While this insight has primarily been derived from relating duplicate gene evolution to expression pattern divergence between different mammalian tissues, we hypothesize that the same patterns will emerge when examining temporal patterns of gene expression across a complex life cycle. To test the predictions that the evolution of duplicate genes corresponds with more divergent and stage-specific expression patterns (Figure 1), we examined patterns of gene expression across the holometabolous life cycle of the monarch butterfly, *Danaus plexippus.* The *D. plexippus* life cycle is characterized by a nondispersive caterpillar stage that is specialized for feeding on milkweed foliage, followed by a nonfeeding pupal stage during which metamorphosis occurs, and a final highly dispersive imaginal (butterfly) stage that is specialized for reproduction and feeding on nectar. The extreme ontogenetic niche shifts and trait divergence between stages make *D. plexippus* a promising model system for studying the evolutionary processes that generate morphological and functional divergence throughout life cycles.

## Methods

### Experimental design and *D. plexippus* rearing

To quantify changes in gene expression across the holometabolous development of *D. plexippus*, we sequenced mRNA extracted from third instars, fifth instars, early pupae (one day after pupation), late pupae (6–8 days after pupation), and adults (several hours after eclosion). A previous study has suggested that feeding on more toxic milkweed induces changes in gene expression during the second instar (Tan et al., 2019). Therefore, we reared larvae on both *Asclepias incarnata* (less toxic) and *Asclepias curassavica* (more toxic) to ensure that our findings are robust to a major source of environmental variation. We collected five individuals at each stage and from each plant for mRNA quantification.

Parental (P) *D. plexippus* butterflies were caught in St. Marks, Florida, USA. (30°09ʹ33″N 84°12ʹ26″W) during October 2022. Butterflies were overwintered in a 14 °C incubator (to maintain a state of diapause) and were fed approximately 10%–20% honey water every 10 days. During March 2023, butterflies were mated to establish an F1 generation. F1 caterpillars were reared on *Asclepias curassavica* and after maturation and mating, the F2 caterpillars used in this experiment were reared on either *A. curassavica* or *A. incarnata.* To reach the necessary sample size, we used F2 caterpillars from two different lineages that did not share P or F1 ancestors. Treatments of plant species and development stage were randomly distributed to caterpillars from both lineages to minimize confounding due to genetic background. All individuals sampled in this study were reared at the same time and in the same conditions (see Supplementary Appendix 1.1–1.3 for details).

### Sample collection and preparation

To minimize the possible effects of sample handling, all caterpillars, pupae, and adults were snap frozen in liquid nitrogen before being stored at −80 °C. Third instars, fifth instars, early pupae, and late pupae were all frozen in sterile centrifuge tubes, and adults were frozen in glassine envelopes several hours after eclosion (after their wings had finished expanding). For each day freezing took place, samples were stored in a polystyrene foam cooler full of dry ice until all flash freezing for that day was completed. This process took approximately 1 h or less on any given day, so no sample was on dry ice for more than an hour before being transferred to the −80 °C freezer. All freezing took place in the same greenhouse room that the caterpillars were reared in, and no individual left said room before being frozen throughout the duration of the experiment.

Because we were interested in global gene expression patterns, we collected samples by homogenizing whole bodies using a sterile porcelain mortar and pestle. Each sample for a given round of homogenization was placed in a cooler filled with dry ice. Samples were individually placed in a mortar, and liquid nitrogen was constantly added throughout the homogenization to prevent samples from thawing. After a given sample was completely homogenized, the homogenate was quickly collected using a sterile polypropylene spatula and stored in a fresh centrifuge tube. Twenty samples were randomly selected for each round of homogenization.

### RNA extraction and sequencing

We used a Promega SV Total Isolation System kit to extract total RNA from *D. plexippus* homogenate. While our workflow generally followed the manufacturer’s suggested protocol, we made several alterations to obtain higher-quality RNA. Briefly, we doubled the recommended RNA lysis buffer, increased the relative centrifugal force in all centrifugation steps, and included an additional centrifugation step to better clear organic contaminants and improve final extract purity. The full RNA extraction protocol used can be found in Supplementary Appendix 1.5. After each extraction, we used a NanoDrop to quantify the purity and concentration of the RNA. Samples with an A260/A280 or an A260/A230 of less than 1.95 were discarded and reextracted. After all extractions were completed, purified RNA was packaged in dry ice and sent to Novogene (Sacramento, CA) for library preparation and sequencing. Briefly, Novogene used an Agilent 5400 Fragment Analyzer System to confirm that all samples had adequate purity levels, concentrations, and volumes, as well as acceptable RNA integrity numbers (minimum = 7.9). Libraries were then prepared via poly-A tail selection and sequenced using a 150 bp paired-end approach on a NovaSeq 6000 sequencing system, thus ensuring at least 20 million reads were obtained for each sample.

### Sequence processing and gene expression quantification

Initial quality control of raw sequences was performed by Novogene, where adapter sequences, reads with ambiguous base calls in greater than 10% of the read, and reads with a phred score of less than or equal to 5 in 50% of the read were removed. After receiving the sequences from Novogene, we used FastQC to generate additional quality reports for each sample (Andrews, 2010). This showed that the median phred score did not drop below 30 at any position for any sample. Therefore, no additional sequence quality control was performed.

To quantify transcript abundances for each gene, we used kallisto (v.0.46.2) to pseudo-align reads to the coding sequences of the *D. plexippus* reference genome (v.Dpv3, GenBank Assembly = GCA_000235995.2) (Zhan et al., 2011). Downstream analyses were performed using transcript per million normalized read counts (automatically generated by kallisto) to minimize biases due to unequal gene lengths and varying library sizes (Abrams et al., 2019; Wagner et al., 2012). Prior to analyses that involved phylogenetic-gene expression comparisons and expression specificity, transcript/million values were log transformed.

### Quantifying gene expression divergence between stages

Our objective was to quantify the overall transcriptional dissimilarity between stages. We used Manhattan distances to quantify this dissimilarity because our data were high-dimensional and because we wanted to consider the magnitudes of transcriptional changes. We first computed the Manhattan distance between each sample using the *dist* R function (R Core Team, 2022). We then used the *adonis2* function from the *vegan* R package (v.2.6-4) (Oksanen et al., 2022) to perform a permutational multivariate analysis of variance (PERMANOVA) with 999 permutations, where developmental stage and plant were initially considered as factors. We then performed a PERMANOVA on each set of adjacent stages, as well as between each larval stage and the adult stage. To visualize global expression divergence between stages, we performed principal coordinate analysis using the *prcomp* R function (R Core Team, 2022).

### Quantifying the relationship between gene phylogenetic divergence and expression pattern divergence within homologous groups

To infer homology between genes, we first used PSI-BLAST (BLAST 2.5.0+) (Altschul, 1997) with five iterations to align all *D. plexippus* protein sequences to each other. Genes were then inferred to be homologous if the query sequence showed at least 30% similarity across the length of the target sequence, as well as an E-value of at least 1 × 10^−10^. To examine how including more distant homologs could impact our analysis, we performed an additional analysis where homology was inferred based on at least 20% similarity across 70% of the target sequence and an E-value of less than at least 1 × 10^−5^. These less stringent cutoffs for homology inference showed consistent results with our primary analysis (Supplementary Appendix 3.2). Homologous pairs were assembled into sets of two-node subgraphs, and subgraphs were then merged based on common node identity to assemble homologous groups.

To quantify the phylogenetic distance between members of inferred homologous groups, we first used MUSCLE (v.5.1) to create a multiple sequence alignment for each group (Edgar, 2004). We then used IQ-TREE2 (v.2.1.4) to identify the best fit sequence evolution model and infer maximum likelihood phylogenies for each multiple sequence alignment (Kalyaanamoorthy et al., 2017; Minh et al., 2020). We then used the *cophenetic.phylo* function from the *ape* R package (v. 5.7-1) (Paradis & Schliep, 2019) to calculate pairwise phylogenetic distances from each homologous group tree, which we note are based on sequence divergence and not inferred divergence time. To calculate pairwise expression pattern distances, we mean centered and standardized the median transcript/million value for each gene across stages by dividing the difference between the transcript/million value and the mean value for each gene by the standard deviation of transcript/million values across stages. This allowed us to better capture temporal trends in expression by minimizing similarities due to expression magnitudes. We then calculated the pairwise Euclidian distance between each gene expression pattern within a given homologous group using the *dist* R function (R Core Team, 2022). Finally, we used Mantel tests to calculate the correlation between phylogenetic and expression pattern distance matrices for each homologous group, which were implemented via the *mantel* function in the *vegan* R package (v.2.6-4) (Oksanen et al., 2022). We then used a *t*-test to test if the distribution of correlation coefficients was positively shifted from 0, which was implemented using the *t-test* R function (R Core Team, 2022).

### Quantifying the relationship between phylogenetic diversity and expression pattern diversity across homologous groups

The diversity (*D*) of each previously described phylogenetic tree was calculated as the sum of branch lengths: $D=\overset{n}{\underset{i=1}{\sum }}l_{i}$, where *n* represents the number of branches and *l*_*i*_ represents the length of the *i*th branch. To quantify expression pattern diversity, we first used the Ward method to create hierarchical clustering graphs of the temporal expression patterns for each gene. Prior to clustering, the transcripts/million values for each gene were mean centered and standardized because hierarchical clustering will group expression patterns that show distinct temporal trends but have more similar average relative abundances across time points. For each hierarchical clustering graph, diversity was calculated as previously described for phylogenetic diversity. All hierarchical clustering graphs were constructed using the *hclust* R function, and all linear models were fit using the *lm* R function (R Core Team, 2022). Our data was nonlinearly related, and both phylogenetic diversity (Shapiro–Wilk test, *W* = 0.717, *p* = 2.909e-16) and expression pattern diversity (*W* = 0.570, *p* < 2.2e-16) were nonnormally distributed. Therefore, we tested that expression pattern diversity monotonically increases with phylogenetic diversity using Spearman’s rank correlations, which were implemented using the *cor.test* R function (R Core Team, 2022). We examined correlations across all homologous groups, as well as within homologous group sizes that had five or more groups, to discern the effects of gene addition and phylogenetic diversification within groups.

### Expression specificity calculation and analysis

Stage-specificity for each gene was calculated using the tissue specificity index τ (Yanai et al., 2005), which ranges from 0 (equal expression across stages) to 1 (expression in a single stage): $\tau =\overset{N}{\underset{i=1}{\sum }}\left(1-x_{i}\right)/N-1$, where *N* is the number of stages (for our purposes) and *x*_*i*_ is the expression level normalized to the maximum expression value across stages. Although τ was developed for assessing tissue specificity, it has been used to gain insight into temporal specificity as well (Cardoso-Moreira et al., 2019). We then performed a Kolmogorov–Smirnov test using the *ks.test* R function (R Core Team, 2022) to assess if the distribution of τ values was shifted in duplicated genes relative to singleton genes.

## Results

### The extent of transcriptional divergence between *D. plexippus* larvae and pupae is comparable to the divergence between larvae and adults

Because all distinct phenotypes expressed throughout a complex life cycle are coded by the same genome, trait decoupling must be mediated through variation in gene expression across stages. Therefore, we were first interested in the extent that gene expression has diverged between stages throughout the *D. plexippus* metamorphosis.

**Figure 2.**
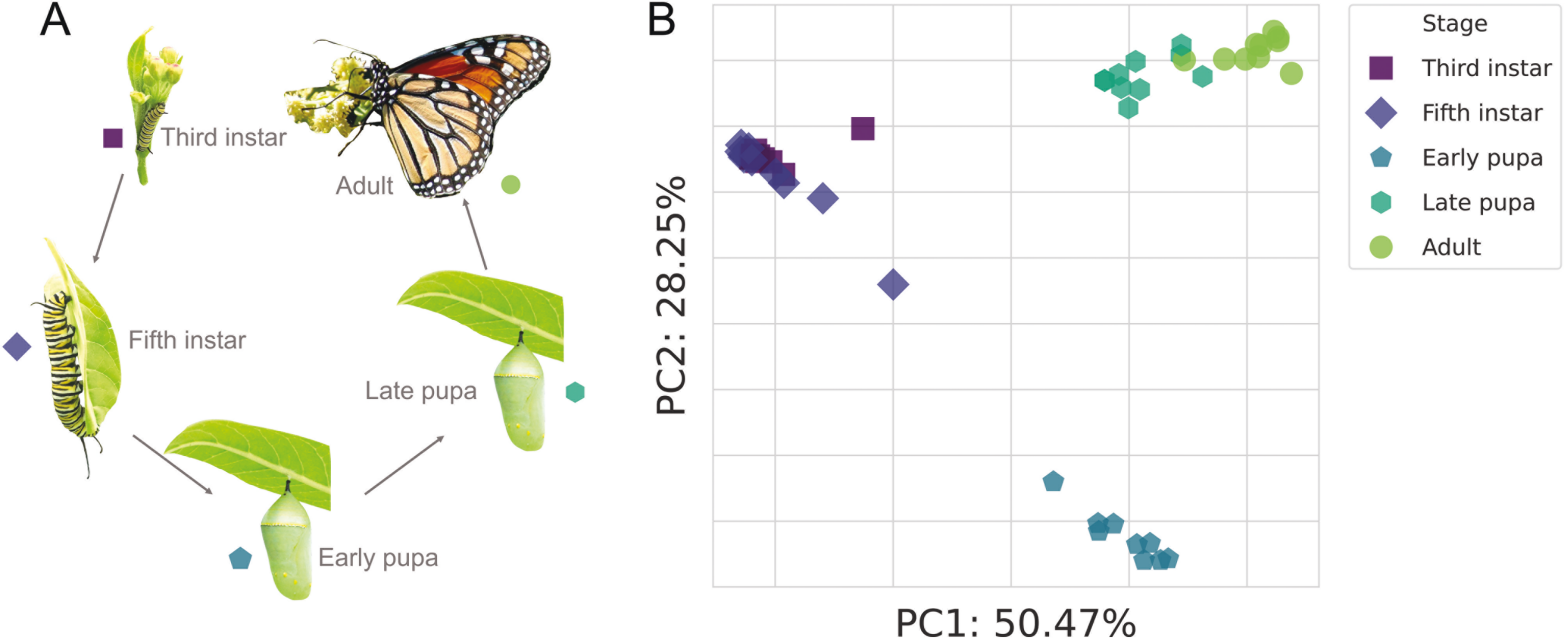
A depiction of how morphology and transcription change across the *D. plexippus* lifecycle. (A) Images of each life stage sampled in this study are shown. (B) A principal coordinate analysis plot showing substantial transcriptional divergence between life stages. Each point represents the global gene expression profile of an individual, and closer points indicate more similar gene expression profiles. Axis labels indicate principal coordinate rank and the proportion of variance explained.

Overall, we found that gene expression significantly varied by developmental stage (*F* = 61.36, *p* < .001) but not plant host (*F* = 0.88, *p* = .47) (Figure 2). We then performed pairwise comparisons to test for differences between subsequent stages as well as between larvae and adults. Following *D. plexippus* throughout metamorphosis: the transition from third instar to fifth instar involves some but relatively few changes in gene expression (distance = 6.97 × 10^5^, *F* = 18.67, *p* < .001). Then a substantial change in gene expression occurs during the transition from fifth instar to early pupa (distance = 1.22 × 10^6^, *F* = 68.06, *p* < .001), followed by a slightly smaller but comparable change from early pupa to late pupa (distance = 1.20 × 10^6^, *F* = 62.25, *p* < .001). Finally, the transition from late pupa to adult involves a modest change in gene expression (distances = 9.37 × 10^5^, *F* = 35.43, *p* < .001), but said change is notably less than the changes involved in the previous two transitions. It is interesting to note that the extent of divergence in gene expression between fifth instars and early pupae is comparable to the divergence between both larval stages and adults (third instar: distance = 1.16 × 10^6^, *F* = 108.08, *p* < .001; fifth instar: distance = 1.24 × 10^6^, *F* = 71.65, *p* < .001). This distinction in early pupae appears to involve a decrease in metabolic investment and an increase in immune investment (Supplementary Appendix Figure S7). More broadly, the transcriptional changes across stages appear to be mostly driven by differential investment in metabolism and genetic information processing, consistent with niche shifting and developmental requirements (Supplementary Appendix Figure S7).

### Phylogenetic divergence between homologs generally corresponds with increased divergence in temporal expression pattern

**Figure 3.**
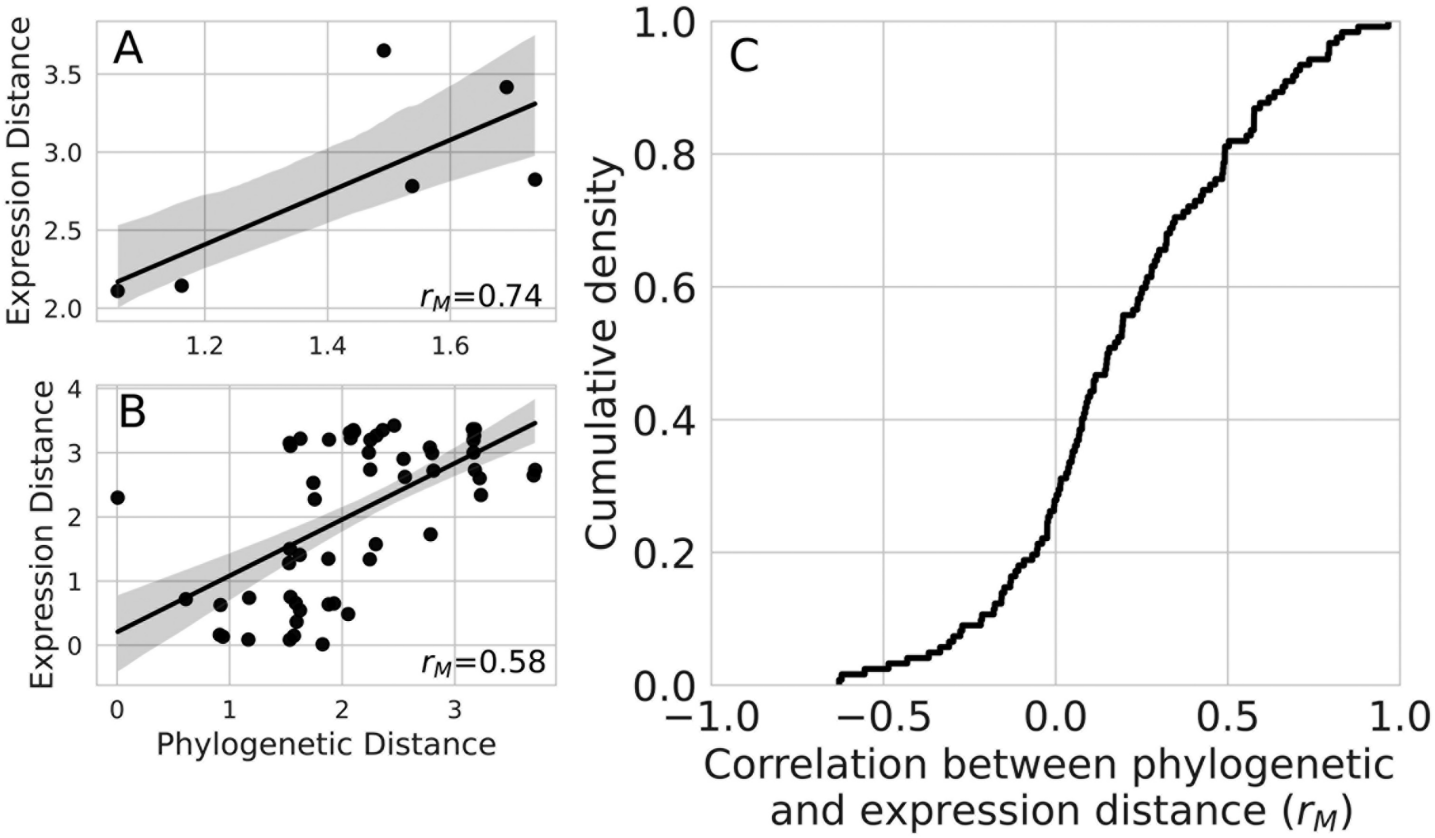
Phylogenetic distance positively correlates with expression pattern distance in most homologous gene groups. The correlation between phylogenetic and expression distances in a set of (A) Hox homologs and (B) Osiris homologs. In (A) and (B), each point indicates the phylogenetic and expression distance for a pair of genes within the homologous groups. A and B are meant to contextualize the broader analysis, not to lend interpretations about the specific homologous groups used for demonstration purposes. (C) The empirical cumulative density function of correlation coefficients between phylogenetic distance and expression pattern distance across all homologous groups. Values greater than 0 indicate a positive correlation and greater values indicate stronger correlations. Overall, the majority of the distribution (approximately 72%) consists of positive correlations.

As previously described, the general hypothesized outcome of evolutionary divergence between homologs, which we measured using phylogenetic distances based on sequence divergence, is increased divergence in their expression patterns. Consistent with this hypothesis, we generally found a positive relationship between phylogenetic distance and expression pattern distance within homologous groups (Figure 3). Specifically, a positive association was observed in approximately 72% of groups. However, we note that there is variation in both the strength and direction of said correlations, with the remaining 28% of groups showing null or negative correlations. Nonetheless, the distribution of correlation coefficients is shifted positively from 0 (mean = 0.19, *t* = 6.29, *p* = 5.38 × 10^−9^).

### Diversity in the temporal expression patterns exhibited by homologous groups increases with their phylogenetic diversity

**Figure 4.**
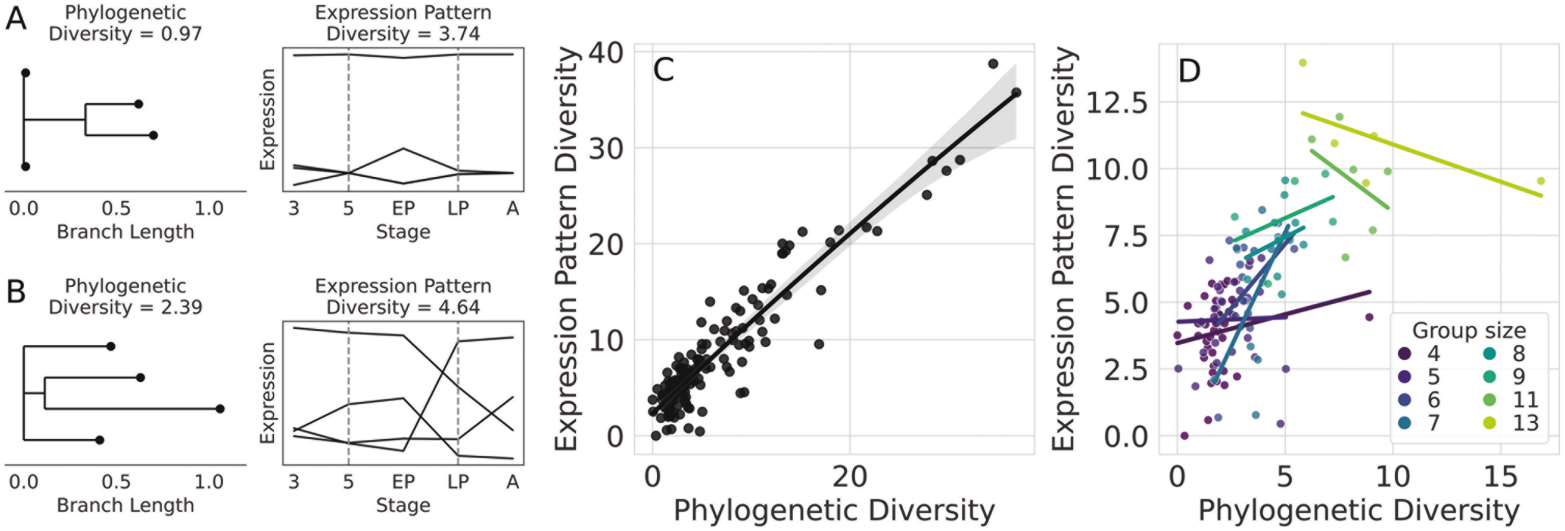
More phylogenetically diverse homologous groups exhibit more diverse patterns of expression. (A) An example of a less phylogenetically diverse homologous group (a geranylgeranyl diphosphate synthase-like group) showing less diverse patterns of expression. (B) An example of a more phylogenetically diverse group (an arrestin-like group) showing more diverse patterns of expression. (A) and (B) are meant to contextualize the broader analysis, not to lend interpretation about the specific homologous groups used for demonstration purposes. (C) The relationship between phylogenetic and expression pattern diversity across all homologous groups. The solid black line depicts the fit quadratic model, and the light gray area indicates the 95% confidence interval for said model. (D) The relationship between phylogenetic and expression pattern diversity by homologous group size. Each line represents the linear model fit to each homologous group size (only group sizes with five or more replicates were considered in this analysis).

If expression patterns diverges with phylogenetic divergence between genes within a homologous group, the predicted emergent pattern is that as a homologous group diversifies (in both size and sequence divergence), the group as a whole should accumulate more different patterns of gene expression. This would result in increased overall expression pattern diversity in homologous groups that are more phylogenetically diverse, which we measured using sequence divergence. Consistent with this hypothesis, we found a positive relationship between phylogenetic diversity and expression pattern diversity (*ρ* = 0.8345, *p* < 2.2e-16) (Figure 4). However, we also found that the increase in expression pattern diversity started to saturate at higher phylogenetic diversities, and that this relationship was better described by a quadratic model than a linear model (linear model SSE = 826.78, quadratic model SSE = 823.52).

The positive relationship between phylogenetic and expression pattern diversity could have been driven by the addition of genes to homologous groups, as opposed to phylogenetic diversification within the group. Therefore, we examined the relationship within each homologous group with five or more replicates. This analysis revealed positive correlations between phylogenetic and expression pattern diversification for each of the six smaller homologous group sizes (mean *ρ* = 0.30, 95% CI = [0.15, 0.45]), where we had statistical power to detect this positive relationship in two out of six homologous group sizes (*p* ≤ 0.0185). However, in the two larger group sizes, we found no associations between phylogenetic and expression pattern diversification (mean *ρ* = −0.52, *p* range = [0.825, 0.8792]). Full results for each group size can be found in Supplementary Appendix Table S4. Overall, these results support a positive but saturating relationship between phylogenetic and expression pattern diversity.

### Genes within duplicated genes tend to show more stage-specific expression patterns than singleton genes

**Figure 5.**
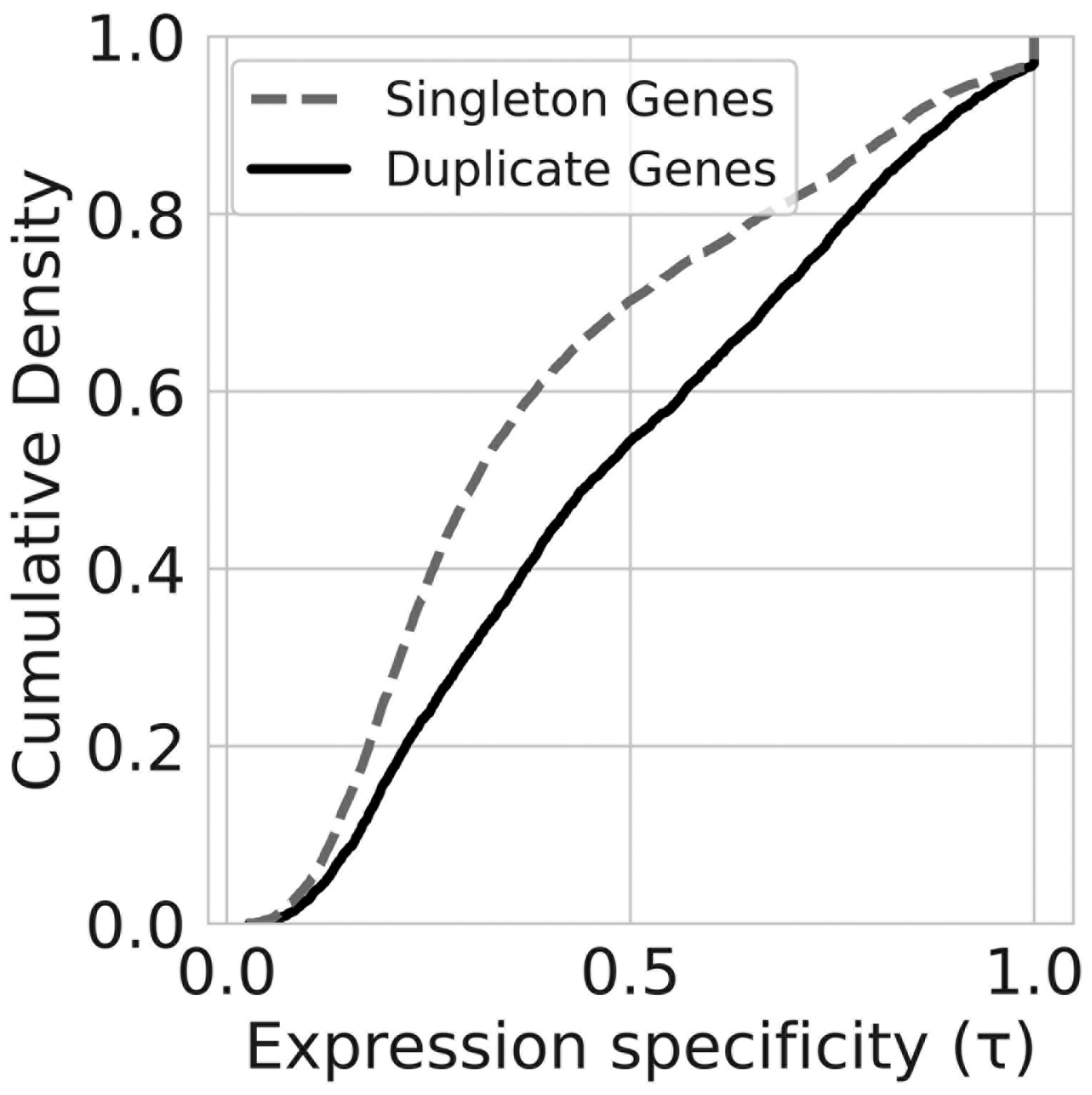
The expression patterns of duplicate genes show increased stage-specificity relative to singleton genes. The empirical cumulative density functions of expression specificity values for duplicate (solid line) and singleton (dashed line) genes. Higher expression specificity values indicate increased stage-specificity.

Another key prediction regarding expression pattern divergence between duplicate genes is that copies will show increased stage-specificity. Consistent with this prediction, we found that genes within homologous groups tended to show increased stage-specificity relative to singleton genes (*D* = 0.193, *p* < 2.2 × 10^−16^) (Figure 5).

## Discussion

Although many studies have investigated the genetic decoupling of traits between life stages, the evolutionary causes and consequences of trait decoupling remain less understood. Therefore, we investigated the link between gene duplication and transcriptional divergence between stages across the *D. plexippus* metamorphosis. By examining how temporal gene expression patterns changed with phylogenetic divergence between duplicate genes, we found that more distantly related genes tended to show more diverged patterns of gene expression (Figure 3). Although the use of pairwise comparisons has been criticized for assessing the relationship between sequence and expression divergence across species (Dunn et al., 2018), as this was not a comparative study across species, distinguishing patterns of divergence between orthologs and paralogs was not central to our goals (duplications that occurred in an ancestral species or more recently could both contribute to trait decoupling between stages). We also found that more phylogenetically diverse groups generally exhibited more diverse patterns of gene expression (Figure 4) and that genes within homologous groups showed increased stage-specificity relative to singleton genes (Figure 5). As predicted, these results are consistent with studies that have examined the role of gene duplication in facilitating expression divergence between different cells and tissues (Cardoso-Moreira et al., 2019; Gu et al., 2002; He & Zhang, 2005; Huminiecki & Wolfe, 2004; Li et al., 2005; Yanai et al., 2005). This consistency suggests that theories of evolution by gene duplication can be applied more generally toward understanding functional differentiation between stages at the organismal level.

Our findings significantly expand on previous findings that duplicate genes were more likely to vary in expression between larvae and prepupae in several *Drosophila* species than singleton genes (Gu et al., 2004). A more nuanced pattern that we observed was a saturating relationship between phylogenetic diversity and expression pattern diversity. This pattern was recapitulated across homologous group sizes, where the positive relationship between expression pattern diversity and phylogenetic diversity disappeared at larger and more diverse groups (Figure 4). Similar patterns have been documented in humans, mice, and yeasts, with expression divergence occurring more rapidly at shorter evolutionary time-scales before plateauing at longer time-scales (Gu et al., 2002; He & Zhang, 2005; Makova & Li, 2003). Possible explanations for this pattern include decoupled rates of evolution in coding sequence and regulatory elements, dosage sensitivities/balancing, and additional complexities related to neo/sub-functionalization dynamics (Papp et al., 2003; Qian & Zhang, 2008; Wagner, 2000, 2001). Regardless of the specific mechanisms, which are beyond the focus of this study, finding this consistency provides stronger evidence that our results recapitulate more fundamental work on duplicate gene evolution. However, deviations from the predicted relationship between phylogenetic divergence and expression pattern divergence were also found. These deviations could likely be explained by the historical context in which specific homologous groups originated and evolved. For example, whether or not the duplication event was lineage-specific or occurred ancestrally, whether or not duplicates arose from a small-scale duplication event or a chromosomal duplication event, and the relative importance of selective and neutral processes in generating sequence divergence are all expected to influence duplicate functionalization and expression divergence (He & Zhang, 2005; Huminiecki & Wolfe, 2004; Makova & Li, 2003).

We interpret our results as evidence for an important link between gene duplication and life cycle evolution. However, it is important to emphasize that we do not suggest that expansion of the specific homologous groups identified in our analyses was directly involved in the origin of holometabolous development; the origin of holometabolous development was not the focus of this study. Rather, our aim was to search for a general process by which traits become temporally decoupled, which would result in greater life cycle complexity when said traits are accumulated over time. Previous studies have documented the patterns that emerge from temporal trait decoupling. Genetic independence of traits expressed by different stages has been well described (see (Aguirre et al., 2014; Cheverud et al., 1982; Goedert & Calsbeek, 2019; Medina et al., 2020) for examples and (Collet & Fellous, 2019) for a detailed review), and more recent studies have elucidated variation in gene expression between stages as the likely cause of said independence (Collet et al., 2023; Critchlow et al., 2019; Herrig et al., 2021; Schott et al., 2022). Our findings are consistent with this interpretation as well. However, a common theme across previous studies is that decoupling is variable and not universal to all traits or genes. Therefore, a more mechanistic understanding of how decoupling evolves is needed to understand life cycle evolution more comprehensively. Our findings suggest a role for gene duplication in the decoupling of traits and, more generally, in facilitating divergence in temporal gene expression patterns across stages.

Although our findings suggest an important link between gene duplication and life cycle evolution, we are not able to make causal inferences because gene duplication is not the only mechanism that facilitates evolutionary change in gene expression patterns. Genes are expressed through regulatory networks, and evolutionary changes to said regulatory elements may be facilitated by, but do not require gene duplication (Wagner, 2001; Zhang et al., 2004). It is possible that the decoupling of traits between life stages is predominately driven by regulatory evolution. Under this hypothesis, the associations we described between sequence divergence and expression divergence could be attributed to regulatory divergence between homologous genes, as opposed to their differential functionalization. Likewise, the regulatory environment of a given stage can shape patterns of stage-specificity in gene expression, which has the potential to influence how duplicate genes evolve. For example, if a gene is expressed ubiquitously across stages, duplication could lead to broad deleterious effects through dosage sensitivity (Papp et al., 2003). Therefore, it is possible that duplicates of genes with stage-specific expression patterns are more likely to be retained, which could explain our observation that duplicate genes show more stage-specific expression patterns. These alternative hypotheses do not necessarily exclude a role of gene duplication in facilitating life cycle evolution, and future studies that aim to quantify their relative importance will lend key insight into the evolution of life cycle complexity. One approach would be to understand how regulatory elements (such as transcription factors) and duplicate genes have evolved across lineages with varying degrees of life cycle complexity.

Because our samples consisted of whole bodies, the variation in gene expression observed between stages likely represents shifts in the relative abundance or activity of different cell and tissue types throughout *D. plexippus* postembryonic development. This, paired with the consistency of our findings with work on the role of gene duplication in generating functional differentiation between cells and tissues, suggests that life cycle evolution in multicellular organisms can be more fundamentally understood through evolutionary shifts in the timing at which different cell and tissue types and functions are expressed. This echoes Haldane’s earlier ideas that changes in the timing at which genes act is an important aspect of evolutionary change (Haldane, 1932). From this perspective, the continuous transition from infant to adult in primates could be mechanistically linked to the extreme transition from larva to butterfly in lepidopterans.

## Supplementary material

Supplementary material is available online at *Evolution Letters*.

qrae028_suppl_Supplementary_Material

## Data Availability

All sequences and count matrices generated for this project have been deposited in the NCBI GEO database and can be accessed with the accession number GSE253389 or the BioProject accession number PRJNA1065445. All code written for data analysis can be accessed at https://github.com/gabe-dubose/mtstp.
