# Supplementary material for: The link between gene duplication and divergent patterns of gene expression across a complex life cycle
Source: Evol Lett. 2024 Jul 2;8(5):726–34. doi: 10.1093/evlett/qrae028 (PMC11424080; doi:10.1093/evlett/qrae028)
Supplement: qrae028_suppl_Supplementary_Material [file qrae028_suppl_supplementary_material.pdf]

Appendix for:
The link between gene duplication and divergent patterns of gene expression across a complex life cycle

James G. DuBose[1,*] and Jacobus de Roode[1]

[1]*Department of Biology, Emory University*
[*]*Corresponding author: James G. DuBose, james.g.dubose@gmail.com*

Contents

1 Extended Methods **2**
1.1 Study system and experimental design 2
1.2 Milkweed cultivation 2
1.3 *D. plexippus* Rearing 2
1.4 Sampling across life stages 3
1.5 RNA extraction and sequencing 3
1.6 Sequence processing and gene expression quantification 4
1.7 Quantifying gene expression divergence between stages 5
1.8 Inferring homologous gene groups 5
1.9 Quantifying the relationship between gene phylogenetic divergence and expression pattern divergence within homologous groups 5
1.10 Quantifying the relationship between phylogenetic and expression pattern diversity 5
1.11 Expression specificity calculation and analysis 6

2 Methodological Summaries **6**
2.1 RNA quality control report 6
2.2 RNA sequencing statistics 7
2.3 Summary of homologous gene group inferences 9

3 Supporting Results **10**
3.1 Analysis of transcriptional divergence using correlation-based distances 10
3.2 Reanalysis based on less stringent homology inference 10
3.2.1 Correlations between phylogenetic and expression pattern diversity 10
3.2.2 Expression pattern specificity 13
3.3 Broad functional overview 13

1 Extended Methods

1.1 Study system and experimental design

Holometabolous development involves the transition from a larval stage that is typically specialized for feeding and growth to a stationary or less mobile pupal stage. During the pupal stage, dramatic morphological restructuring occurs, resulting in a distinct adult stage that is typically specialized for dispersal and reproduction.

To quantify changes in gene expression the across the holometabolous development of *Danaus plexippus*, we sequenced mRNA extracted from third instars, fifth instars, early pupae (one day after pupation), late pupae (6-8 days after pupation), and adults (several hours after eclosion). A previous study has suggested that feeding on more toxic milkweed induces changes in gene expression during the second instar [1]. Therefore, we reared larvae on both *Asclepias incarnata* (less toxic) and *Asclepias curassavica* (more toxic) to ensure that our findings are robust to a major source of environmental variation. We collected five individuals at each stage and from each plant for mRNA quantification. All individuals sampled in this study were reared at the same time and in the same conditions.

1.2 Milkweed cultivation

A. incarnata and *A. curassavica* seeds were purchased from Joyful Butterfly (Blackstock, SC, USA). To break cold dormancy, seeds were placed in sand-filled bags and kept at 4°C for two months prior to sowing. Approximately two months before the start of the experiment, seeds were sown into Lambert LM-GPS germination soil and placed in a temperature-controlled greenhouse room that was held between 25°C and 29.4°C. *A. incarnata* germination rates tend to be relatively low, so seed trays were topped with vermiculite to aid in moisture retention. Seedlings were fertilized with approximately 20 PPM of Jack's LX 15-5-15 with 4% Ca and 2% Mg fertilizer three times a week until the majority of plants grew two sets of true leaves. All plants were then re-potted into Pro-mix BK25 soil, moved to a new temperature-controlled room that was held between 25.6°C and 29.4°C, and fertilized three times a week as described above. Approximately one week before the start of the experiment, plants were moved into the same greenhouse room that caterpillars were reared in (described below).

1.3 *D. plexippus* Rearing

Monarch butterflies were caught and labeled near St. Marks, Florida, U.S.A. (30°09'33"N 84°12'26"W) between October 21st and October 23rd, 2022. Clear tape was placed on the abdomen of each butterfly and examined under a stereomicroscope to ensure they were not infected by *Ophryocystis elektroscirrha*, a common parasite of monarch butterflies. Prior to mating season, wild-caught monarch butterflies were stored in glassine envelopes at 14°C to induce a state of diapause, and were fed approximately 10-20% honey water every 10 days. Between March 6th and March 15th, 2023, wild-caught monarchs were placed in mesh cages for mating. Each cage was set up in a climate-controlled growth chamber (25°C, 16-hour/8-hour day/night cycle) and contained three male and three female butterflies. All cages were provided with a petri dish containing a sponge soaked in approximately 10-20% honey water for butterfly feeding. Mating cages were checked every 14 hours, and copulated butterflies were transferred to their own separate cage. After a copulated pair had detached the next day, the male was removed from the cage and the female was given a potted *A. curassavica* plant for oviposition, as well as honey water as described above. After a given female was done laying eggs, the plant was taken out of the growth chamber and placed in a temperature-controlled greenhouse room that was held between 23.3°C and 27.8°C for.

F1 caterpillars were reared on *A. curassavica* in the same greenhouse room previously described. After pupation, the silk attached to the end of the pupal cremaster was used to hot glue the pupae to the lid of clear solo cups, which were then taken from the greenhouse to the laboratory (22°C) for eclosion. A piece of paper towel was placed in the bottom of cups to help absorb liquids produced during the eclosion process. After eclosion, butterflies were placed in glassine envelopes and stored as previously described.

Between April 23rd and May 1st, 2023, F1 butterflies from different lineages and that were not infected with *O. elektroscirrha* were mated as previously described in the F0 generation. F1 females were given either *A. curassavica* or *A. incarnata* for oviposition, and caterpillars were collectively placed on their treatment

plant species upon hatching. Care was taken to make sure caterpillars that had taken bites of the plant they were oviposited on to were placed on the same milkweed species. Likewise, only caterpillars that had not taken any bites of the plant they were oviposited on were placed on the other milkweed species. To reach the sample size needed for this experiment, we used F2 caterpillars from two different lineages that did not share F0 or F1 ancestors. Treatments of plant species and development stage were randomly distributed to caterpillars from both lineages to minimize confounding due to genetic background.

1.4 Sampling across life stages

To minimize changes in transcription due to sample handling, all caterpillars, pupae, and adults were snap frozen in liquid nitrogen before being stored in -80°C. Third instar caterpillars were pulled from their feeding plant and quickly placed into a sterile 2mL microcentrifuge tube that was then dipped in liquid nitrogen. Fifth instar caterpillars were frozen in the same way but were placed in sterile 5mL centrifuge tubes. Caterpillars that ate all of the leaves off of the plant they were originally placed on were placed on another plant of the same species.

One day after pupation, early pupae were placed in 5mL centrifuge tubes and frozen in liquid nitrogen as described above. Three days after pupation, pupae assigned to late pupa and adult stages were removed from their plant and taped to the lids of clear solo cups using silk attached to the cremaster. In some cases, not enough silk detached with the pupa, and tape was applied directly to the cremaster. Solo cups were then placed on the bottom rack of the same shelf that the caterpillars were reared on, and shade was provided by placing plastic trays above and to the southeast facing side of the shelf to prevent pupae from burning. A piece of paper towel was placed in the bottom of the cups containing adult samples to absorb fluids produced during the pupation process. Since there is variation in how long it takes for a pupa to eclose, late pupae were collected 6-8 days after pupation. Care was taken to ensure the distributions of how many days after pupation late pupae were sampled were equal between plants. Adults were frozen several hours after eclosion to allow their wings to fully expand. Here, adults were removed from their solo cup and quickly placed in glassine envelopes, which were then quickly frozen in liquid nitrogen.

After flash freezing in liquid nitrogen, samples were stored in a styrofoam cooler full of dry ice until all freezing for that day was completed. This process took approximately one hour or less on any given day, so no sample was on dry ice for more than an hour before being transferred to the -80°C freezer. All freezing took place in the same greenhouse room that the caterpillars were reared in, and no monarch left said room before being frozen throughout the duration of the experiment.

1.5 RNA extraction and sequencing

We use a Promega SV Total Isolation System kit to extract total RNA from the monarch homogenate. Extractions were performed in batches of 11 samples with 1 negative control (per extraction batch). After each extraction, we used a NanoDrop to quantify the purity and concentration of the RNA. Samples with an A260/A280 or an A260/A230 of less than 1.95 were discarded and re-extracted to meet purity standards. While the general workflow followed the manufacturer's suggested protocol, we made some alterations to obtain higher quality RNA extract. Briefly, we doubled the recommended RNA lysis buffer to decrease the tissue concentration in the initial lysis step. All centrifugation steps were increased to 20,000 rcf to better remove organic contaminants and performed at 17°C to avoid sample heating. We also added an additional centrifugation step after the initial tissue lysis to further clear organic contaminants and improve final extract purity. The specific protocol is as follows:

1. Add homogenate to 2mL microcentrifuge tube.
2. Immediately add 590 uL of RNA Lysis Buffer (RLA+BME) into microcentrifuge tube with homogenate
3. Use sterile micropestle to crush and lyse monarch homogenate (vigorously crush and spin pestle in tube for approximately 1 minute).
4. Centrifuge at 20,000 rcf for 10 minutes at 17°C.
5. Transfer approximately 400 uL to 500 uL of aqueous phase to a new microcentrifuge tube.

6. Centrifuge at 20,000 rcf for 20 minutes at 17°C.
7. Transfer 175uL of the cleared lysate (aqueous layer) to a new microcentrifuge tube.
8. Add 200 uL of 95
9. Transfer lysate+ethanol to Spin Basket Assembly and centrifuge for 5 minutes at 20,000 rcf and 17°C.
10. While the centrifuge is running, Prepare DNase incubation mix: 40uL of Yellow Core Buffer + 5uL MnCl2 + 5uL of Dnase I (per sample). Mix gently via pipetting.
11. Discard eluate.
12. Add 50 uL of DNase incubation mix to the membrane of the Spin Basket, incubate for 15 minutes at room temperature.
13. Add 200 uL of DNase Stop Solution (DSA+ethanol) and centrifuge at 20,000 rcf for 1 minute at 17°C.
14. Discard eluate.
15. Add 600 uL of RNA Wash Solution (RWA); centrifuge at 20,000 rcf for 1 minute at 17°C.
16. Discard eluate.
17. Add 250 uL of RNA Was Solution (RWA); centrifuge at 20,000 rcf for 2 minutes at 17°C.
18. Transfer Spin Basket to Elution Tube.
19. Add 100 uL of Nuclease-Free water to the Spin Basket membrane.
20. Centrifuge at 20,000 rcf for 1 minute to elute RNA.
21. Store at -80°C.

After all extractions were completed, purified RNA was packaged in dry ice and sent to Novogene for sequencing. Briefly, Novogene used an Agilent 5400 Fragment Analyzer System to performed additional quality control. This involved reconfirming sample purity, ensuring that all samples had adequate concentrations and volumes, and checking that all sampled had acceptable RNA integrity numbers (minimum = 7.9). After additional quality assessment, mRNA was separated via poly-A tail selection, and 150bp paired-end sequencing was performed using a NovaSeq 6000 sequencing system, ensuring at least 20 million reads were obtained for each sample.

1.6 Sequence processing and gene expression quantification

Quality control of raw sequences was initially performed by Novogene. This entailed the removal adapter sequences, the removal of reads with ambiguous base calls in greater than 10% of the read, and the removal of reads with a phred score of less than or equal to 5 in 50% of the read. After receiving the sequences from Novogene, we used FASTQC to generate additional quality reports for each sample [2]. This showed that for each sample, the median phred score did not drop below 30 at any position along the reads. Therefore, no additional quality control was performed.

To quantify transcript abundances for each gene, we used kallisto (v.0.46.2) to pseudo-align reads to the coding sequences of the D. plexippus reference genome (v.Dpv3; GCA_000235995.2) [3]. Downstream analyses were performed using transcript per million normalized read counts (automatically generated by kallisto) to minimize biases due to unequal gene lengths and varying library sizes [4, 5].

1.7 Quantifying gene expression divergence between stages

Given the high dimensionality of gene expression data, we first computed the Manhattan distance between each sample using the dist R function [6]. We then used the adonis2 function from the vegan R package (v.2.6-4) [7] to perform a permutational multivariate analysis of variance (PERMANOVA) with 999 permutations, where developmental stage and plant were initially considered as factors. We then performed a PERMANOVA on each set of adjacent stages, as well as between each larval stage and the adult stage. To visualize global expression divergence between stages, we performed principal component analysis using the *prcomp* R function [6]. To ensure our findings were robust to different metrics for evaluating overall transcriptional differences, we performed the same analysis using Pearson correlation distances, which were calculated using the *cor* R function [6].

1.8 Inferring homologous gene groups

To infer homology between genes, we first used PSI-BLAST (BLAST 2.5.0+) [8] with five iterations to align all D. plexippus protein sequences to each other. Genes were then inferred to be homologous if the query sequence showed at least 30% similarity across the length of the target sequence, as well as an E-value of at least 1x10-10. To examine how including more distant homologs could impact our analysis, we performed an additional analysis where homology was inferred based on at least 20% similarity across 70% of the target sequence and an E-value of less than 1x10-5. Homologous pairs were assembled into sets of two-node subgraphs, and subgraphs were then merged based on common node identity to assemble homologous groups.

To quantify the phylogenetic distance between members of inferred homologous gene groups, we first used MUSCLE (v.5.1) to create a multiple sequence alignment for each group [9]. We then used IQ-TREE2 (v.2.1.4) to identify the best fit sequence evolution model and infer maximum likelihood phylogenies for each multiple sequence alignment [10, 11].

1.9 Quantifying the relationship between gene phylogenetic divergence and expression pattern divergence within homologous groups

To quantify the relatinship between phylogenetic divergence and expression divergence within homologous groups, we used the *cophenetic.phylo* function from the *ape* R package (v. 5.7-1) [12] to calculate pairwise phylogenetic distances from each homologous group tree. To calculate pairwise expression pattern distances, we first mean centered and standardized the median transcripts/million value for each gene within each stage to better measure distance between temporal patterns as opposed to magnitude (which cannot be assessed with our data). We then calculated the pairwise Euclidian distance between each gene expression pattern within a given homologous group using the *dist* R function [6]. Finally, we used Mantel tests to calculate the correlation between phylogenetic and expression pattern distance matrices for each homologous group, which were implemented via the *mantel* function in the vegan R package (v.2.6-4) [7]. We then used a t-test to test if the distribution of correlation coefficients was positively shifted from 0, which was implemented using the *t.test* R function [6].

1.10 Quantifying the relationship between phylogenetic and expression pattern diversity

The diversity (D) of each tree was then calculated by summing all branch lengths: $D = \sum_{i=1}^{n} l_i$, where n represents the number of branches and l_i represents the length of the *ith* branch. To quantify expression pattern diversity, we first created hierarchical clustering graphs of the temporal expression patterns for each gene using the Ward method, as implemented by hclust R function [6]. Prior to clustering, the transcripts/million values for each gene were mean centered and standardized because hierarchical clustering will group expression patterns that show distinct temporal trends but have more similar relative abundances at each time point. For each hierarchical clustering graph, diversity was calculated as previously described for phylogenetic diversity. We then fit a linear model to examine the relationship between phylogenetic diversity and expression pattern diversity across all inferred homologous gene groups, which was implemented using the *lm* R function [6]. Because diversity was calculated additively (for each branch, diversity was added in

proportion to divergence), we also fit individual linear models to each homologous gene group size that had at least five replicates. In addition to removing the inherent positive correlation between group size and diversity, this approach also allowed us to contrast global and local patterns.

1.11 Expression specificity calculation and analysis

Stage-specificity for each gene was calculated using the tissue specificity index τ [13], which ranges from 0 (broad expression) to 1 (specific expression): $\tau = \frac{\sum_{i=1}^{N}(1-x_i)}{N-1}$, where N is the number of stages (for our purposes) and x_i is the expression level normalized to the maximum expression value across stages. Although τ was developed for assessing tissue specificity, it has been used to gain insight into temporal specificity as well [14]. We then performed a Kolmogorov–Smirnov test using the *ks.test* R function [6] to assess if the distribution of τ values was shifted in duplicated genes relative to singleton genes.

2 Methodological Summaries

2.1 RNA quality control report

Table S1: RNA extract quality control report.

Sample Name	Concentration (ng/ul)	Volume (ul)	Total amount (ug)	RIN
mtstp3cu2	120.15	91	10.93	9.7
mtstp3cu3	435.84	93	40.53	9.6
mtstp3cu4	99.07	88	8.72	9.6
mtstp3cu5	223.96	94	21.05	9.7
mtstp3cu8	111.95	91	10.19	9.6
mtstp3iu81	211.09	92	19.42	9.8
mtstp3iu82	194.64	93	18.1	9.8
mtstp3iu83	373.28	91	33.97	9.6
mtstp3iu84	196.04	93	18.23	9.6
mtstp3iu85	136.84	90	12.32	9.7
mtstp5cu17	363.74	101	36.74	9.4
mtstp5cu18	236.69	92	21.78	9.8
mtstp5cu19	544.95	93	50.68	9.5
mtstp5cu20	130	91	11.83	9.7
mtstp5cu21	400.58	94	37.65	9.8
mtstp5iu100	377.26	91	34.33	9.7
mtstp5iu101	98.09	89	8.73	9.6
mtstp5iu97	164.64	92	15.15	9.8
mtstp5iu98	258.2	91	23.5	9.8
mtstp5iu99	139.84	91	12.73	9.8
mtstpAcu65	132.79	92	12.22	9.5
mtstpAcu66	106.76	90	9.61	9.5
mtstpAcu67	37.05	94	3.48	8.9
mtstpAcu68	94.26	91	8.58	9.4
mtstpAcu69	176.26	91	16.04	9.8
mtstpAiu145	162.84	92	14.98	9.6
mtstpAiu146	283.45	92	26.08	9.6
mtstpAiu147	51.58	92	4.75	9.2
mtstpAiu148	89.37	92	8.22	9.3
mtstpAiu149	307.63	91	27.99	9.7
mtstpEcu33	203.48	92	18.72	7.4
mtstpEcu34	244.57	91	22.26	8.7

Sample Name	Concentration (ng/ul)	Volume (ul)	Total amount (ug)	RIN
mtstpEcu35	491.84	89	43.77	7.9
mtstpEcu36	220.03	92	20.24	8.6
mtstpEcu38	220.92	93	20.55	8.6
mtstpEiu113	333.32	93	31	8.9
mtstpEiu114	446.98	91	40.68	7.9
mtstpEiu115	182.76	92	16.81	8.6
mtstpEiu116	257.7	89	22.94	8.4
mtstpEiu117	326.41	92	30.03	8
mtstpLcu49	95.18	92	8.76	9
mtstpLcu50	93.51	92	8.6	9.3
mtstpLcu52	220.52	93	20.51	8.8
mtstpLcu53	95.67	91	8.71	9.4
mtstpLcu56	399.94	89	35.59	8.2
mtstpLiu129	145.89	91	13.28	9.1
mtstpLiu130	91.59	91	8.33	8.7
mtstpLiu131	391.6	87	34.07	9.6
mtstpLiu133	85.42	89	7.6	8.4
mtstpLiu135	108.7	92	10	8.1

2.2 RNA sequencing statistics

After quantifying transcript counts per gene, we checked that our sequencing effort was adequate to downstream analyses. First, we examined the number and proportion of raw reads that passed quality control, as well as the number and proportion of quality-controlled reads that were pseudo-aligned to the *D. plexippus* genome (Table 2). We then generated a rarefaction plot see if our sequencing depth had sufficiently detected the expression of most transcripts that were expressed at a given stage (Figure 1).

Table S2: Sequence processing and mapping summary.

Sample	Raw PE reads	Passed QC	% Passed QC	Pseudo-aligned	% Pseudo-aligned
mtstp3cu2	21943709	2132928515	97.2	16582965.404739982	0.777474036
mtstp3cu3	22924309	2202796852	96.09	17321415.999555275	0.786337423
mtstp3cu4	24873976	2408547096	96.83	18718506.832656555	0.777170057
mtstp3cu5	27818742	2729296778	98.11	21155266.226326376	0.77511784
mtstp3cu8	21942370	2133237211	97.22	16603108.222044216	0.778305766
mtstp3iu81	25932842	2532860678	97.67	19868699.32840216	0.784437119
mtstp3iu82	22375940	2195750992	98.13	17106242.600410897	0.779061135
mtstp3iu83	23183162	2267545075	97.81	17444460.402408678	0.769310414
mtstp3iu84	23107037	2261023570	97.85	17945429.764851093	0.793686099
mtstp3iu85	21374435	2100251983	98.26	15988137.614267953	0.761248543
mtstp5cu17	24827892	2419229796	97.44	19638683.30078156	0.811774199
mtstp5cu18	23283921	2277633152	97.82	18962699.68340757	0.832561629
mtstp5cu19	20552516	1987839348	96.72	17087863.371912975	0.859619938
mtstp5cu20	22934654	2237734191	97.57	18115485.776358116	0.809545917
mtstp5cu21	23666289	2302019931	97.27	19702178.064739898	0.855864791
mtstp5iu100	31121258	3038368419	97.63	25782153.591624036	0.848552579
mtstp5iu101	21768932	2121164734	97.44	18187966.46325012	0.85745186
mtstp5iu97	23849683	2315565722	97.09	19239689.897892967	0.830885071
mtstp5iu98	21766794	2105284316	96.72	17675440.16384149	0.83957497
mtstp5iu99	23386986	2292860107	98.04	18686179.461194277	0.814972505
mtstpAcu65	23267178	2266921153	97.43	17092435.515985236	0.753993384
mtstpAcu66	26977628	2628700072	97.44	20072649.61273931	0.763596038
mtstpAcu67	24972220	2433542839	97.45	18546857.73716618	0.762134015
mtstpAcu68	22515202	2194331587	97.46	15951574.031512374	0.726944557
mtstpAcu69	21267172	2062915684	97	15662285.557752984	0.759230524
mtstpAiu145	29683116	2870950980	96.72	23068781.60188841	0.80352405
mtstpAiu146	22849815	2210262605	96.73	17606493.53499071	0.796579261
mtstpAiu147	22404070	2200079674	98.2	16188725.539826853	0.735824513
mtstpAiu148	25214198	2456367169	97.42	18462578.21672617	0.751621274
mtstpAiu149	22156963	2154321512	97.23	16710014.455382776	0.775650912
mtstpEcu33	22763953	2217664301	97.42	16862537.346874774	0.760373756
mtstpEcu34	25629454	2499384354	97.52	18633231.46842286	0.745512848
mtstpEcu35	22465556	2195109477	97.71	16842284.066866323	0.767263968
mtstpEcu36	22055561	2159239422	97.9	16253631.989667526	0.75274802
mtstpEcu38	23213904	2279605373	98.2	17463986.10284253	0.7660969
mtstpEiu113	23868417	2301870135	96.44	17119222.41677067	0.743709306
mtstpEiu114	22466254	2186865164	97.34	16425515.425250849	0.751098682
mtstpEiu115	23622008	2313067023	97.92	17237434.840021413	0.74521986
mtstpEiu116	33296616	3242424466	97.38	23547226.785158955	0.726222832
mtstpEiu117	22703490	2224942020	98	17101827.281335317	0.76864148
mtstpLcu49	27550907	2684560378	97.44	21053416.44129032	0.78424075
mtstpLcu50	27343921	2665485419	97.48	21414551.829342157	0.803401575
mtstpLcu52	26550778	2588435347	97.49	20277129.22413158	0.783373989
mtstpLcu53	21114739	2069033275	97.99	15824607.707317442	0.764830991
mtstpLcu56	36034764	3517353314	97.61	27879259.42907198	0.792620386
mtstpLiu129	20400384	1985773379	97.34	15447977.51483457	0.777932552
mtstpLiu130	34495919	3368181531	97.64	26660707.28941579	0.791546033
mtstpLiu131	22712422	2196972580	96.73	17262201.926361006	0.785726781
mtstpLiu133	22825856	2225064443	97.48	17685552.79521903	0.794833285
mtstpLiu135	24991444	2433166988	97.36	19590944.360775467	0.805162344

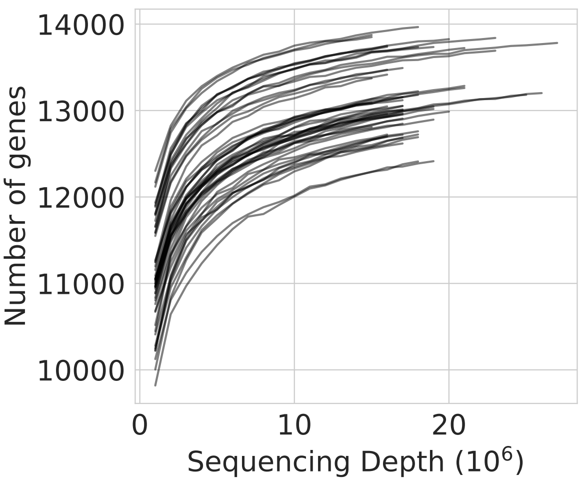

Figure S1: Rarefaction curves showing the number of genes detected on the y-axis and sequencing depth on the x-axis. Each line corresponds to an individual sample. Plateaus in the number of detected genes at higher sequencing depths suggest that our sequencing effort was sufficient.

2.3 Summary of homologous gene group inferences

Homologous gene group inference is described in section 1.8. The following plots show the summary histograms of homologous group size.

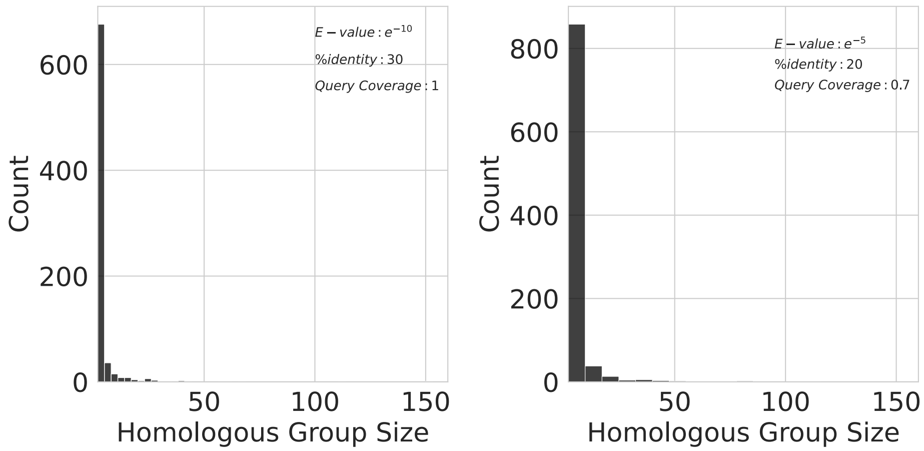

Figure S2: Histograms showing the distribution of homologous group sizes detected in the *D. plexippus* genome when using more (left) and less (right) stringent sequence similarity cutoffs.

Table S3: A table showing the number of duplicate and singleton genes identified in this study.

PSI-BLAST Parameters	n Homologs	n Singletons
e-value = $e^{-}10$, %identity = 30, query coverage = 1	3237	11995
e-value = $e^{-}5$, %identity = 20, query coverage = 0.7	9792	5440

3 Supporting Results

3.1 Analysis of transcriptional divergence using correlation-based distances

To ensure that our inference of transcriptional divergence between stages was robust, we performed an additional analysis using Pearson distances, as opposed to Manhattan distances (presented in the main text). We found that expression significantly varied by developmental stage ($F = 194.94$, $p < 0.001$) but not plant ($F = 0.87$, $p = 0.37$) (Figure S3). We then performed pairwise comparisons to test for differences between subsequent stages, as well as between larvae and adults. Following *D. plexippus* throughout metamorphosis: the transition form third instar to fifth instar involves some, but relatively few changes in gene expression ($F = 40.84$, $p < 0.001$). Then a substantial change in gene expression occurs during the transition from fifth instar to early pupa ($F = 182.74$, $p < 0.001$), followed by another substantial change from early pupa to late pupa ($F = 274.12$, $p < 0.001$). Finally, the transition from late pupa to adult involves another substantial change in gene expression ($F = 231.28$, $p < 0.001$). It is interesting to note that the difference between third instar and adults ($F = 458.90$, $p < 0.001$) is comparable to the difference between third instar larvae and early pupae ($F = 453.98$, $p < 0.001$). Likewise, the difference between fifth instar larvae and adults ($F = 217.05$, $p < 0.001$) is comparable to the difference between fifth instar larvae and early pupae. These findings are consistent with the analysis based on Manhattan distances, and highlight the same interesting point that pupae are approximately as transcriptioanlly diverged from larvae as adults are.

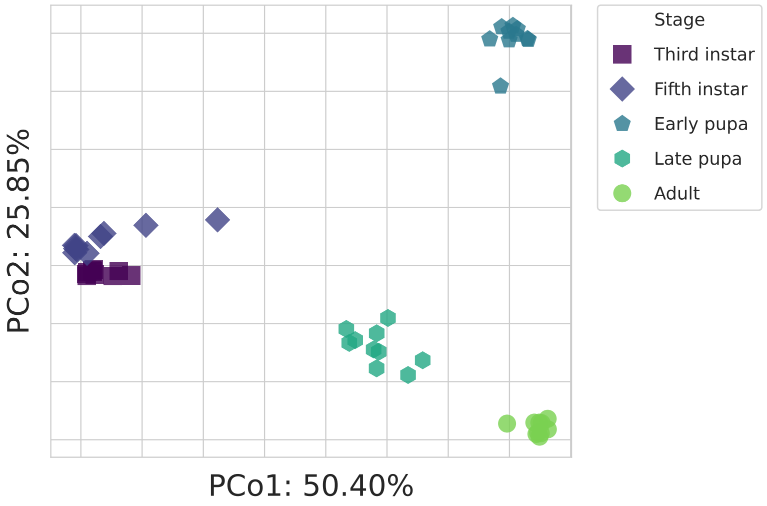

Figure S3: A principal coordinate analysis plot showing substantial transcriptional divergence between life stages. Each point represenst the global gene expression profile of an individual, and closer points indicate more similar gene expression profiles. Axis labels indicate principle coordinate rank and the proportion of explained variance.

To visualize of the lack of an effect of plant on transcriptional divergence, we also created a visualization of the Manhattan-distance based PCoA (Figure S4).

3.2 Reanalysis based on less stringent homology inference

To ensure that our findings were robust, we re-analyzed our data using less stringent sequence identity cutoffs to infer gene homology (see section 1.8). This included more divergent genes in our analysis, thus increasing the amount of phylogenetic diversity captured.

3.2.1 Correlations between phylogenetic and expression pattern diversity

The overall relationships between phylogenetic and expression pattern diversity were consistent with our primary analysis (Figure S5).

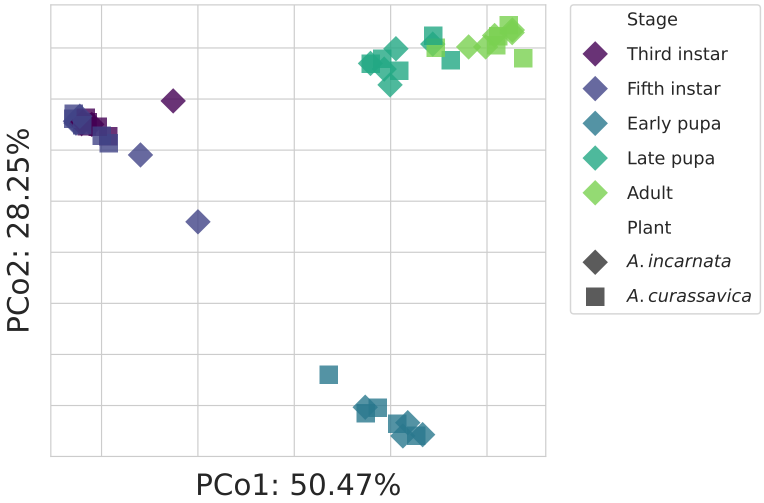

Figure S4: A principal coordinate analysis plot showing substantial transcriptional divergence between life stages, and a lack of differentiation based on which plant larvae were reared on. Each point represents the global gene expression profile of an individual, and closer points indicate more similar gene expression profiles. Axis labels indicate principle coordinate rank and the proportion of explained variance.

Table S4: A table showing the results for each correlation between phylogenetic and expression pattern diversity that were summarized in the main text.

Homologous group size	ρ	p-value
Total	0.8345044	$< 2.2e-16$
4	0.3434008	0.01234
5	0.18	0.1938
6	0.1178571	0.3382
7	0.6454545	0.0185
8	0.3212121	0.1838
9	0.2	0.3917
11	-0.5428571	0.8792
13	-0.5	0.825

Table S5: A table showing the results for each correlation between phylogenetic and expression pattern diversity that were produced by our reanalysis based on less stringent similarity cutoffs for homology inference.

Homologous group size	ρ	p-value
Total	0.8268648	$< 2.2e-16$
4	0.3319884	0.0007816
5	0.3084583	0.02998
6	0.03387097	0.4282
7	0.3169231	0.0614
8	0.6363636	0.02722
9	0.3818182	0.1395
10	0.04242424	0.4593
12	-0.6	0.8833
14	-0.5	0.825
16	0.3	0.3417

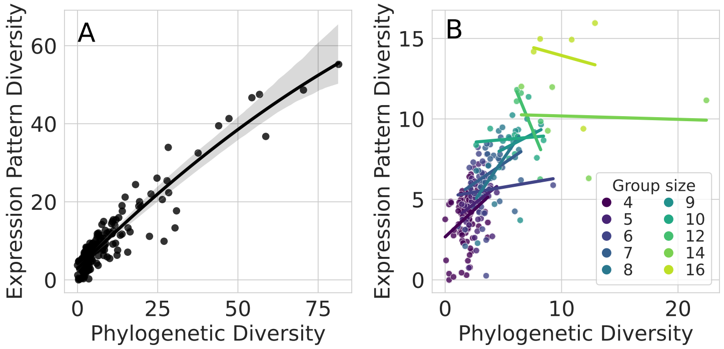

Figure S5: The relationship between phylogenetic and expression pattern diversity A) across all homologous gene groups, B) within each homologous gene group size. In A, the solid black line depicts the fit polynomial model, the light gray area indicates the 95% confidence interval for said model. In B, each line represents the linear model fit to each homologous group size (only group sizes will 5 or more replicates were considered in this analysis)

3.2.2 Expression pattern specificity

Our comparisons of stage-specificity between duplicate and singleton genes based on less stringent similarity cutoffs for homology inference were consistent with the analysis presented in the main text. Specifically, genes that are part of homologous groups tend to show increased stage-specificity relative to singleton genes (D = 0.13, $p < 2.2 * 10^{-16}$). Although this pattern is statistically supported, we note that the effect size is slightly smaller than the analysis presented in the main text analysis.

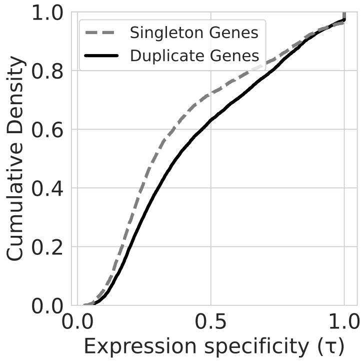

Figure S6: The empirical cumulative density functions of expression specificity values τ for duplicate (solid line) and singleton (dashed line) genes. Higher expression specificity values indicate more stage-specific expression patterns.

3.3 Broad functional overview

To gain a general sense of what high level functional differences occurred between stages, we used the KEGG [15] to infer gene functions and examined the relative transcriptional investment in the highest level KEGG BRITE groupings (Figure 6). Genes that were classified to multiple high-level KEGG categories were excluded from the analysis for more conservative estimates of functional investments.

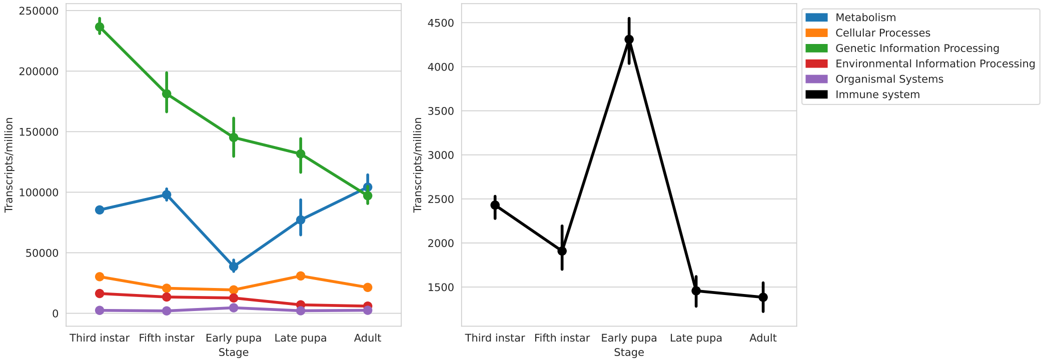

Figure S7: A line plot showing the relative transcriptional investment (transcripts per million) in each high level functional group across life stages. Note that these groupings reflect the overall transcriptional investment in each broad functional group listed, not the activity of individual pathways or genes. Therefore, each individual pathway or gene within each group is not expected to necessarily follow the exact trend exhibited by the whole group. Error bars represent 95% confidence intervals calculated across individual samples.

References

[1] W Tan, et al., Transcriptomics of monarch butterflies (*Danaus plexippus*) reveals that toxic host plants alter expression of detoxification genes and down-regulate a small number of immune genes. *Molecular Ecology* **28**, 4845–4863 (2019).

[2] S Andrews, FastQC: A Quality Control Tool for High Throughput Sequence Data. (2010).

[3] S Zhan, C Merlin, J Boore, S Reppert, The Monarch Butterfly Genome Yields Insights into Long-Distance Migration. *Cell* **147**, 1171–1185 (2011).

[4] ZB Abrams, TS Johnson, K Huang, PRO Payne, K Coombes, A protocol to evaluate RNA sequencing normalization methods. *BMC Bioinformatics* **20**, 679 (2019).

[5] GP Wagner, K Kin, VJ Lynch, Measurement of mRNA abundance using RNA-seq data: RPKM measure is inconsistent among samples. *Theory in Biosciences* **131**, 281–285 (2012).

[6] R Core Team, *R: A Language and Environment for Statistical Computing.* (R Foundation for Statistical Computing, Vienna, Austria), (2022).

[7] J Oksanen, et al., *vegan: Community Ecology Package.* (2022).

[8] S Altschul, Gapped BLAST and PSI-BLAST: a new generation of protein database search programs. *Nucleic Acids Research* **25**, 3389–3402 (1997).

[9] RC Edgar, MUSCLE: multiple sequence alignment with high accuracy and high throughput. *Nucleic Acids Research* **32**, 1792–1797 (2004).

[10] BQ Minh, et al., IQ-TREE 2: New Models and Efficient Methods for Phylogenetic Inference in the Genomic Era. *Molecular Biology and Evolution* **37**, 1530–1534 (2020).

[11] S Kalyaanamoorthy, BQ Minh, TKF Wong, A Von Haeseler, LS Jermiin, ModelFinder: fast model selection for accurate phylogenetic estimates. *Nature Methods* **14**, 587–589 (2017).

[12] E Paradis, K Schliep, ape 5.0: an environment for modern phylogenetics and evolutionary analyses in R. *Bioinformatics* **35**, 526–528 (2019).

[13] I Yanai, et al., Genome-wide midrange transcription profiles reveal expression level relationships in human tissue specification. *Bioinformatics* **21**, 650–659 (2005).

[14] M Cardoso-Moreira, et al., Gene expression across mammalian organ development. *Nature* **571**, 505–509 (2019).

[15] M Kanehisa, S Goto, KEGG: Kyoto Encyclopedia of Genes and Genomes. *Nucleic Acids Research* **28** (2000).
